# *"We noticed that suddenly the country has become full of MRI"*. Policy makers' views on diffusion and use of health technologies in Iran

**DOI:** 10.1186/1478-4505-8-9

**Published:** 2010-04-06

**Authors:** Mohammad Palesh, Carol Tishelman, Sten Fredrikson, Hamidreza Jamshidi, Göran Tomson, Azita Emami

**Affiliations:** 1 Division of Global Health (IHCAR), Department of Public Health Sciences, Karolinska Institutet, Sweden; 2 National Public Health Management Center (NPMC), Tabriz University of Medical Sciences, Tabriz, Iran; 3 Kermanshah University of Medical Sciences, Kermanshah, Iran; 4 Research and Development Unit, Stockholms Sjukhem Foundation, Stockholm, Sweden; 5 Medical Management Center (MMC), Department of Learning, Informatics, Management and Ethics (LIME), Karolinska Institutet, Sweden; 6 School of Nursing, Midwifery and Social Work, University of Manchester, UK; 7 Division of Neurology Huddinge, Department of Clinical Neuroscience, Karolinska Institutet, Sweden; 8 Department of Pharmacology, Faculty of Medicine, Shaheed Beheshti University of Medical Sciences, Tehran, Iran; 9 College of Nursing, Seattle University, Seattle, USA

## Abstract

**Objective:**

Uncontrolled proliferation of health technologies (HT) is one contributor to the increasing pressure on health systems to adopt new technologies. With limited resources, policy-makers encounter difficulties in fulfilling their responsibility to meet the healthcare needs of the population. The aim of this study is to explore how policy-makers' reason about the diffusion and utilization of health technologies in Iran using magnetic resonance imaging (MRI) and interferon beta as tracers.

**Method:**

This qualitative exploration complements quantitative data generated in a research project investigating the diffusion and utilization of MRI and interferon beta in Iran. Qualitative semi-structured interviews were conducted with 13 informants in different positions and levels of authority in the Ministry of Health (MOH), University of Medical Sciences, Health Insurance Organizations, and Parliament. The data was analysed using the framework approach.

**Findings:**

Although policy-makers appeared to be positive to health technology assessment (HTA), the processes of policy-making described by the interviewees did not seem to be based on a full understanding of this (discipline). Several obstacles to applying knowledge about HT and HTA were described. The current official plan for MRI adoption and diffusion in the country was said not to be followed, and no such plan was described for interferon beta. Instead, market forces such as advertising, and physician and consumer demand, appear to have strong influence on HT diffusion and use. Dual practice may have increased the induced demand and also reduced the supervision of the private sector by the MOH.

**Conclusion:**

Management instability and lack of coordination in the MOH were found to be important obstacles to accumulation of knowledge and experience which, in turn, could have led to suboptimal managerial and policy-making processes. Furthermore marketing should be controlled in order to avoid creating unnecessary patient demands and negative influences on physicians' behavior.

## Introduction and Aim

Health technologies (HT) are essential tools for addressing health problems. They function as the backbone of all health systems [1], regardless of the degree of sophistication of the system. Health systems are under increasing pressure to adopt new technologies due to rapid and continuous innovation. The increasing number of emerging HTs serves to intensify both patients' and physicians' demands. Levin and colleagues [2] argue that open-ended introduction of HTs in health systems can lead to unrealistic expectations and excessive demands.

Low- and middle-income countries (LMICs) face increasing challenges due to incorporation, diffusion and utilization of new and modern HTs. In a one-way flow of HTs from high-income countries to LMICs, the relevance of HTs to importing countries has often been neglected in lieu of 'newness' of HTs [3]. It is suggested that proliferation of HTs is a major contributor to rising healthcare costs [4,5]. With limited resources, health authorities and policy-makers encounter difficulties in fulfilling their responsibilities in meeting the health needs of the population. Policy-makers therefore need a systematic approach for efficient allocation of resources. While the aim of health technology assessment (HTA) is to provide scientific evidence to guide healthcare decisions and policy-making [6], many LMICs, which are now exposed to HT in the health market, lack government and/or private organizations for HTA [7].

In our previous work, we have examined the diffusion and utilization of two technologies--magnetic resonance imaging (MRI) and interferon beta--considered to be both sophisticated and costly in one middle-income country, Iran [8-10]. These technologies have provided new horizons in diagnostics and therapeutics, making them captivating for many health systems. However, they also provide examples of the challenges for most low- and middle-income countries to afford new expensive technologies with limited public resources. Magnetic resonance imaging (MRI) is a medical imaging device used to visualize detailed internal structures of the body. Because of MRI's ability to make great contrast between the different tissues of the body, it is widely used for diagnostic purposes especially in neurological and musculoskeletal imaging. MRI uses no ionizing radiation and imaging can be performed without injecting contrast agents. It is a safe and highly effective imaging technology. MRI was introduced in the late 1970s [11].

Interferon beta is a drug which is mainly used for treatment of multiple sclerosis (MS). There are three interferon beta products (Avonex, Rebif and Betaferon) which have been used in Europe and the US over the past decade. It has been shown to be an effective drug in reducing relapse rates in MS [12]. All three types of interferon beta have been available in Iran since 1995.

Herceptin is a medicine for treating women with breast cancer whose tumors are HER2 (Human Epidermal growth factor Receptor 2) positive [13]. HER2 status determines how aggressive the breast cancer is and what treatments are most likely to be effective [14]. Because of the prognostic value of HER2 protein, testing for it, performed on cancer cells removed during breast biopsy or surgery, is becoming more common. To receive Herceptin, the tumor must be tested and be HER2 positive [14].

Findings from our earlier studies indicate that, through unplanned diffusion, both MRI and interferon beta have spread rapidly in Iran. Interferon beta has been found to be used at levels higher than those in some high-income countries [10].

In order to better clarify and understand these previous findings, we conducted an exploratory qualitative study to investigate how policy-makers' reason about the diffusion and utilization of health technologies in Iran using MRI and interferon beta as tracers.

## Background

### Study context

The Islamic Republic of Iran is a middle-income country located in Southwest Asia, in the Middle East region. Demographic, economic, and health indicators of Iran are shown in Table 1.

**Table 1:** Demographic, Economic and Health Worker Indicators for Iran

Population (million) (2006) ^a^	70
GDP/capita (International $, 2004)^a^	8367
Total expenditure on health as % of GDP (2005) ^a^	7.8
Total expenditure on health per capita (International $, 2005)^a^	677
Life expectancy at birth, male/female (2005) ^a^	69/73
Physicians/10,000 inhabitants (2005) ^a^	9.0
Hospital beds/10,000 population (2005) ^b^	17.2

a: World Health Organization. Accessed January 2009. http://www.emro.who.int/index.asp

b: World Health Organization. Accessed January 2009. http://www.who.int/en/

GDP, gross domestic product

Healthcare in Iran is provided by both a public (government) sector and a private sector, with health insurance organizations (HIO) also involved in financing healthcare provision. At the national level, the Ministry of Health (MOH) is responsible for policy-making, planning, financing and supervision. On the provincial level, medical universities have some responsibility for financing and planning. At the district level, urban and rural health centers and district hospitals are executive units. Although the importance of primary healthcare is emphasized and financed by the MOH, the Ministry of Health also has an important role in providing hospital care. Secondary and tertiary healthcare is financed through insurance schemes.

The situation in Iran is such that, in accordance with the Iranian National Drug Policy [15], all pharmaceuticals offered in the national market should meet quality criteria satisfying international and national standards. Only those drugs registered in the National Drug List (NDL) are produced in, or imported to, Iran. Drugs on this list are selected by the Iranian Drug Selecting Committee (IDSC), under the Food and Drug Department of the MOH. Members of the IDSC rely on information provided by the expert team of the IDSC secretariat when determining whether or not to include a new formula in the NDL [16].

On the other hand, medical devices and equipment are the domain of the Directorate General of Medical Equipment under the Deputy Minister for Health Affairs. This directorate determines the need for procuring equipment [17]. As noted by the WHO, while some components of technology planning and managing do exist in Iran, others are said to be neglected or immature and are not linked to form a cohesive whole [17].

## Methods

This qualitative explorative study complements other data generated in a comprehensive multi-method, multi-disciplinary research project investigating the diffusion and utilization of HTs in Iran. In this study, semi-structured interviews were conducted with participants with experience of national or provincial policy-making on healthcare. This method is particularly appropriate when quantitative research has been carried out and qualitative data is required to clarify and illustrate the meaning of findings [18].

Eighteen informants were purposefully [19] selected to obtain a variation in professional roles and agency affiliations in relation to HT. These individuals had roles at the MOH, Universities of Medical Sciences (UMS), the main HIOs and Parliament. After receiving permission from the Ethics Committee of the University of Medical Sciences of Kermanshah, Iran, potential informants were contacted by telephone and asked to suggest a time at which an interview could be conducted. Ultimately, we were able to complete 13 interviews with informants in different positions and levels of authority in the MOH, UMS, HIO, and Parliament as shown in Table 2. One person declined participation in the study; practical and logistic reasons prohibited the participation of others.

**Table 2:** Number of interviewees selected, their position and number of interviews conducted

Position	No. of purposefully selected interviewees	No. of interviews conducted
Ministry of Health	8	7
University of Medical Sciences	3	1
Health Insurance Organization	4	4
Parliament	3	1

Total	18	13

### Data Collection

The research team formulated an interview guide with topics to be included in semi-structured interviews with the interviewees. The interview guide was designed to encourage the interviewees to describe their actual experiences with HT in conversational form and to generate discussion of their perspectives on the topics selected.

The first author (MP) conducted and audio-taped interviews, after receiving written informed consent from the 13 informants. The interviews began with an open question to invite the respondents to relate their recent experiences in decision-making related to HT. Follow-up questions further probed areas of interest raised in the course of conversation with the interviewees. Use of MRI and interferon beta functioned as examples of HT when probing during the interviews. Topics addressed included views on: type of information needed for decision-making, experiences of technology dissemination in Iran including factors that facilitate and obstruct dissemination, factors that might facilitate or obstruct technology utilization, and policy needs regarding technology dissemination and use.

The audio-taped interviews which were conducted in Farsi lasted approximately 50 minutes and were later transcribed verbatim by a professional transcriber and checked for consistency with the recording by the first author. The three team members who were fluent in Farsi validated analyses of all interviews. For further discussion and validation, two transcripts were translated into English to make them accessible to the members of the research group who were not proficient in Farsi. Finally, quotations selected from all interviews were translated for the manuscript.

### Data analysis

A framework approach [20,21] was used for analysis of the interview texts. This approach was developed at the National Centre for Social Research in England in the 1980s for use in applied qualitative research with clear policy or clinical aims. This analytic method facilitates linking qualitative and quantitative data [22].

**Figure 1:**
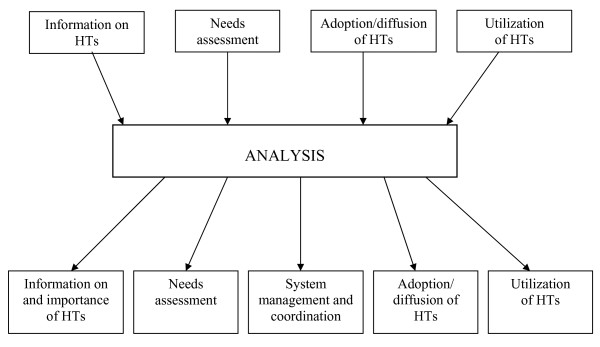
**Description of the analytic process**.

The data was analyzed using the following stages of framework analysis. To achieve general understanding, the transcripts were read repeatedly by (MP) the first author and (AE) the last author *(familiarization with data)*. In the next stage a thematic framework was developed to function as the coding scheme *(thematic analysis)*. Based on existing literature and reviews of the interviews, preliminary categories were developed. The categories were then re-evaluated and rephrased during the analytical process (Fig 1). Transcripts were then coded *(indexing) *by (MP) the first author and (AE) the last author. All codes derived from each transcript were transferred to a specific reference sheet which contained the number of the transcript, page number from which the code was drawn, and number assigned to that specific code. In the next stage, *charting*, data was restructured by thematic content. In order to allow tracking back to the interview source, each theme, together with code related, was recorded on a new sheet using the identifiers given above. Charts were used to define and illustrate concepts and experiences explicated by the participants *(mapping and interpretation)*.

Quotes which exemplify findings are presented in italics with the interview number shown in parentheses. Information that might identify the policy-maker is excluded, in an effort to ensure confidentiality.

## Results

The major themes are presented in Fig. 1. These themes, presented here separately for clarity, are interrelated, and consist of the following: *information about and importance of HTs; needs assessment; system management and coordination; adoption/diffusion of HTs; utilization of HTs*. The themes were addressed from various perspectives by the informants, for example with regard to their own opinions, descriptions of praxis and, to a lesser extent, regulations.

### Information about and importance of HTs

Almost all interviewees pointed to the importance of HTs and their utilization. They stated that HTs were a means through which science and knowledge could be implemented, whilst, at the same time, recognizing that treatment of disease has become increasingly more complex through the influence of HTs. One interviewee explained that:

*"Health technologies are of great importance when physicians are unable to formulate a diagnosis based on physical examination, for example, when an affected organ does not have any pain receptors that could signal the pain, or when the affected organ is not directly accessible to physicians" *(Int #7).

Several interviewees pointed out that HTs assist physicians in providing their patients with sophisticated treatment, with one interviewee explicitly emphasizing the limitations a specialist would face without HTs, saying: "*A physician without health technology is like a soldier without a gun" *(Int #9).

Several interviewees argued that HTs reduce treatment costs and that the ultimate costs of a system are also decreased when applying new HTs such as innovative surgical procedures, since they enable patients to recover quickly and return to work. The potential role of HTs for improving health was described by one of the interviewees as follows:

*"For the Third World countries, sometimes such preliminary activities as plumbing works, sanitary services or vaccination, which we know as primary healthcare, leave much more impact than technologies of advanced healthcare on promotion of health in society. This means that primary health care is a priority for a country where the infant mortality rate is three hundred out of one hundred thousand. But when a country shows an infant mortality rate of less than thirty, it means that this country has good primary health care, and now for the purpose of reducing such a rate from thirty to fifteen, it needs to use such other specialized services such as advanced technology and drugs" *(Int #10).

In contrast to the many positive comments, several interviewees described HTs as not only as constructive, but also potentially destructive if used in an uncontrolled manner: "*health technologies could potentially be harmful to public health. By consuming financial resources health technologies will impede public health promotion and preventive activities" *(Int #13).

There was a high degree of consensus among interviewees that importers or producers of HTs were their main sources of information about HTs. For patients, however, physicians were said to be the direct source of information about HTs: "*There is an information asymmetry in the health market, people are not so informed and physicians are those who produce the information" *(Int #5).

Interviewees argued that informing patients about HTs could produce an increased demand for HTs. Disseminating information was deemed important in the sense that correct information could lead to appropriate demand, but incorrect information could result in inappropriate demand. One interviewee said: "*Inhabitants of a very small district wanted to be provided with an MRI machine; they thought that MRI is a therapeutic device" *(Int #3). This view was supported by an interviewee from another agency who said: "*When I go to rural areas all people want me to provide them with doctors and not other health workers" *(Int #6).

Several interviewees clarified that data about HTs is not systematically collected, processed and compiled. They claimed that data is not usually used for proper decision-making and allocation of resources. One of the interviewees said that *"unaware authorities would not be able to inform people and manage technologies correctly" *(Int #13).

### Needs assessment

Lack of needs assessment was considered by interviewees from two dimensions. The first dimension was whether any specific type of technology is needed in the health system as articulated by *"It is said that Herceptin is not allowed to be imported into France but our market is open to that drug and we use it excessively" *(Int #4). Several interviewees stated that HTs are imported into the country without any planning. They pointed out that import of HTs is not usually based on needs assessment and structured planning, resulting in a wide variety of HTs in the health system. Several interviewees pointed out that as inclusion of many HTs is not the result of real needs assessment, they can cause problems such as unnecessary use and increased treatment costs. This view was articulated by one of the interviewees: "*There is a close relationship between MRI availability in the private sector and increasing unnecessary use of MRI for patients" *(Int #11), while another said:

*"We actually spend a lot of money for MS patients but it should be noted that interferon beta is not the only treatment that MS patients need. They may also need rehabilitation, psychological and social support" *(Int #8).

Another interviewee pointed out the increasing demand for importing MRIs, despite the charge for MRI examinations remaining the same for the past four years. This was seen to indicate that the tariff was high enough to provide a substantial profit. Physicians were also said to use HTs as soon as they are introduced, without paying enough attention to needs assessment. This, in turn, was said to lead to the general population's increasing awareness of HTs as a potential for raising or inducing demand.

A second dimension raised concerning needs assessment was the extent to which a technology should be incorporated into the health system. As one interviewee said: "*...the number of MRI machines which had been installed in Tehran province was enough for the whole population of the country" *(Int #11). Several interviewees stated that wealthier Western countries are able to use very costly HTs like portable and mobile MRI machines, but are then able to deliver services with fewer machines, questioning why Iran does not adopt such a policy.

One interviewee stated that performing needs analysis in relation to newly introduced HTs is ignored especially when similar technologies are already in use in the health system. However, two interviewees from another agency claimed that, in the case of pharmaceuticals, HTs will not be imported into the country unless they are more effective and less expensive than those already in Iran's pharmaceutical market. Several interviewees emphasized that regardless of needs assessment, when new technology is imported into the country, it easily substitutes previous HTs.

One specific aspect of needs was addressed by an interviewee who emphasized that HTs are needed for medical students' training even if their cost benefits are not proven.

A few interviewees pointed to the scarcity of experts and skillful persons for performing needs assessment both in the MOH and HIOs. Training such experts to perform needs analysis is a task which was seen as important. Interviewees described different levels of proficiency available for different types of assessments. They also differed in their evaluations of this. For example, two interviewees explicitly referred to all drugs being assessed with regard to safety, quality and other pharmacological and pharmacoeconomic aspects, with needs assessment performed by expert committees. While one of them stated needs assessment is based on needs in the community and the burden of disease, the other said: "*we would not consider the number of patients *(when determining) *whether or not the drug is needed in Iran" *(Int #10).

One interviewee claimed that, although health is a multidisciplinary issue, the lack of attention to multidisciplinary perspectives results in a lack of comprehensiveness in identifying needs and needs assessment. It was also brought up that HT needs are judged in relation to the level of health of the community, i.e. the higher the level of health, the more advanced and sophisticated the technology which is seen to be necessary. As one interviewee said: *"You know, I think that under some conditions we can say that its role (HTs) is extensive, and under some other conditions, we can say that its role is not much. From my viewpoint, it depends on society" *(Int #10).

### System management and coordination

Interviewees raised the lack of structured policy-making in relation to HTs. Health system managers were described as not getting involved in HT management. Weakness in planning and policy-making was repeatedly mentioned by interviewees. One interviewee stated that *"Less attention is paid to the importance of health management as a necessary expertise and skill for managing the health system. There is a limited amount of research on the health system and health policy in Iran" *(Int #6). Another interviewee supported this view, saying: *"There is a lack of a strong expert body in the Ministry of Health for decision-making on health technologies" *(Int #5). One interviewee linked management to healthcare costs and quality of care by saying: *"We are not certain that increasing healthcare costs, because of using health technologies, has resulted in improving the quality of care. It means that when we import technologies we need to know how to manage them" *(Int #2).

Weaknesses in applying management information systems to determine policy and allocation of resources, coupled with lack of attention to non-medical evidence (social and economic) were pointed to as contributing to poor planning.

Another factor addressed was management instability, which led to loss of accumulated experience. In an unstable situation when policy-makers are frequently changed, it would be difficult to make devised policies sustainable, as stated by this interviewee: *"...the Cabinet is changed periodically every four years. Ministers and other senior officials are replaced. This unstable executive situation is an impediment to the accumulation of experience" *(Int #7) and *"when managers are dismissed they take their experience with them" *(Int #13). Such management problems result in other health system problems, as articulated by two interviewees from different agencies: *"We have enough financial resources, our problem is in managing them which encounters us with financial restrictions" *(Int #4), and *"lack of precise planning on how to apply health technology, causes financial constraints in the Ministry of Health" *(Int # 6).

According to one interviewee: "*In developing countries, although public opinion is not very strong and because of weakness in governmental management, it is the market which finally holds the dominant position" *(Int #7). This statement was supported by two other interviewees from two other agencies, who said: *"Under the pressure of physicians and beneficiary demands, we have not had alternatives other than changing our policies in relation to health technologies" *(Int #4), and *"we knew that what the community wanted was a false demand but, because of their high pressure, we were not able to control it" *(Int #12).

Another issue raised related to detrimental effects of physicians working simultaneously in public and private sectors (dual practice). Some interviewees pointed to lower salaries and income in the public sector compared to the private sector as a major incentive for dual practice. Negative effects of dual practice which were addressed included: a shift of patients from the public to the private sector, facilitating induced demand, and reducing the ability of government agencies to maintain control, as stated by one interviewee: "*The Ministry of Health and Universities of Medical Sciences do not have an efficient overview due to some public executive managers practicing in the private sector" *(Int #5).

Lack of coordination as an obstacle to HT management was also addressed by interviewees, e.g. *"Many attempts were made to institute coordination in the Ministry of Health but, unfortunately, all attempts failed. Because each deputy in the Ministry of Health considers himself to be in the position of authority, coordination is a difficult task to be achieved" *(Int # 6). Another interviewee stated that *"We have scattered islands, each organization decides by itself; health insurance by itself, the Medical Association by itself and the Ministry of Health by itself" *(Int #10). According to one interviewee, the large number of HIOs also serves as an obstacle to coordinating HT-related policies.

### Adoption/diffusion of HTs

According to two interviewees, although the expert team should assess each new drug from various perspectives before including it in the NDL, pharmacoeconomic assessment is not performed for all drugs since it is both time consuming and complicated. As stated:

*"The pharmacoeconomic committee does not assess all drugs but only those drugs which are expensive or that are claimed could be used for intractable diseases" *(Int # 10).

These interviewees stated that IDSC members are usually pressured by drug importers and local manufacturers to include their formulas in the NDL. Interviewees emphasized that one important criterion for adopting pharmaceuticals is that they have already been approved by agencies like the FDA and have European or the US marketing permission.

Other interviewees clarified that the sovereign authority for decision-making on HT financial coverage is the Medical Services Insurance Supreme Council, composed of representatives from different organizations, ministries and major HIOs. This authority was said to make coverage decisions based on information on cost-effectiveness and cost-benefit analysis. One interviewee said that *"... to cover a service *(HT), *health insurance organizations rely on cost-effectiveness rather than clinical effectiveness, because a health insurance organization is an economic corporation with limited resources and a large number of beneficiaries" *(Int #4).

Although the importance of conducting technology assessment was emphasized, an interviewee from a different agency claimed that HIOs do not select appropriate technologies because they do not perform such assessments. The drawbacks of lacking HTA were emphasized by an interviewee who said *"we would not pay enough attention to cost-effectiveness and cost-benefits of health technologies, that's why we are so passive when adopting health technologies" *(Int #8).

Several interviewees said that import of technologies into the country and adoption without structured criteria and control is increasing, as articulated by two interviewees: *"we noticed that suddenly the country has become full of MRI" *(Int #5); *"it is said that Iran is the second largest importer of new technologies among Middle-East countries" *(Int #3). Lack of rules and regulations; weak planning in relation to whether and to what extent an HT should be imported or not; the large and active private sector in the HT market and the inability of the MOH to control the HT market, especially in the private sector, all contribute to current problems according to interviewees.

Despite many such statements, other views were also expressed. One interviewee claimed that the need for HTs, exemplified by MRIs, has been identified with national distribution determined by the stratification scheme, which is a structured plan for distribution of health facilities, including HTs, throughout the country. This scheme is based on provincial and district needs estimated by the MOH.

The role of physicians in adoption and diffusion of HTs was noted from several perspectives, as indicated by statements such as: *"Physicians always would like to apply innovations for their likely curative effects on patients" *(Int #1), and *"Physicians encourage and persuade district, provincial and national authorities to adopt health technologies, in fact they want to use technologies wherever they are. That's why they influence diffusion of health technologies" *(Int #13).

A factor often referred to by interviewees was the influence on physicians of HT importer/manufacturer advertising. One interviewee exemplified this by saying: *"Physicians are the most influential factor for adopting health technologies. It is very likely that advertisements by health technology producers stand behind them" *(Int #13).

Other factors mentioned as influencing decisions on the adoption and diffusion of HTs included the economic situation, size of population and coverage by health insurance. Geographical inequities in HT distribution were addressed both in relation to provincial and regional levels. Differences in how readily HT uptake occurred in different provinces was said to be related to problems in health system performance in relation to equity. One interviewee said that *"One important factor in technology adoption which makes provinces different is the ability of their authorities and politicians to obtain facilities from the government and the Ministry of Health" *(Int #12). This view, which focused on the provincial level, can be contrasted with another interviewee's perspective, which focuses on technology adoption at the regional level: "*... another influential factor is the intention of authorities of the country who want to develop their country and make it famous by adopting sophisticated and advanced technologies to attract patients from neighboring countries" *(Int #13)

### Utilization of HTs

Most interviewees said that HT utilization is increasing in the country, although without regulation by a specific plan or guidelines. Inappropriate use of technologies was said by these interviewees to be a major problem in Iran.

As noted above, this was often related to the behavior of physicians. A wide range of other contributors to the unnecessary use of HTs was also described, including uncontrolled importation of HTs, high tariffs, lack of guidelines, and the use of HT to increase profits. The important potential role that guidelines could play for appropriate use of HTs was also emphasized.

Interviewees from different agencies stated that a large amount of interferon beta is used in Iran, with use increasing in recent years. Two interviewees stated that this use is not proportional to the expected increase in MS patients as there has been a 10-fold increase in interferon beta use over the past six years. As is the case with other HTs, one interviewee related the increasing use of interferon beta to advertising by pharmaceutical companies. Another reason described was physicians' practice, as some rely on MRI results rather than clinical findings. This was said to lead to inappropriate use of interferon beta. Other factors for increased interferon beta use mentioned by interviewees were the increasing number of MS patients, incremental consumption of the drug even in cases without indication, and government subsidies making it affordable for more MS patients. One interviewee said that: *"During the last one-two years, we attempted to adjust the subsidy of this drug because we believe that this drug is not really useful for the patients in most of the cases. For some patients, however, it has been useful" *(Int #10).

Prescribing drugs without adequate indication is problematic, as stated by an interviewee: *"Herceptin is supposed to be used only by those patients who are receptor positive. Although most of our clinical laboratories cannot technically detect the receptor, physicians prescribe this drug to many patients" *(Int #4).

## Discussion

Although policy-makers appeared to be positive to Health Technology Assessment (HTA), the processes of policy-making described by the interviewees did not seem to be based on a full understanding of this (discipline). Several obstacles to applying knowledge on HT and HTA were described. The current official plan for MRI adoption and diffusion in the country was said not to be followed, and no such plan was described for interferon-beta. Instead, market forces such as advertising, and physician and consumer demand, appear to have strong influences on HT diffusion and use.

These findings were made possible through the use of semi-structured interviews, analyzed using framework analysis, to better understand policy related issues of health technology in a middle-income country. Health technology and its assessment, despite its history in Western countries, is a new field of research in some middle-income countries [23]. At the same time qualitative research approaches are still considered innovative in this field. Strengths of this study include it being designed to provide answers to questions which emerged from empirical quantitative studies conducted by the same researchers. Combining different areas of expertise in healthcare, health policy and research allowed the research team to investigate these issues comprehensively [24]. Although the sample is a limited one, and a wider range of perspectives might have added additional information, this data is unique and provides considerable insight into policy-makers' perspectives of the factors obstructing the development of systematic HTA decision-making in Iran.

It has been suggested that "policy develops through evolution rather than revolution and the wise policy-maker makes incremental changes to reduce uncertainty and avoid mistakes" [25]. Furthermore, HTA has been pointed out as a form of research to support the process of policy-making in healthcare by providing reliable information, which can be utilized in formulating and addressing problems [26,27]. Thus, it is important to understand why evidence is not readily transferred into practice in the policy-making process. However, as Black [28] argues, to be influential in the policy-making process, research should target the values of policy-makers.

One finding was that needs assessment was not a well established process in procuring HTs. Furthermore, scarcity of trained and skillful experts was described as one obstacle to performing needs assessment. Policy-makers need context-specific input to fit the particular purpose [25]. Obviously, one requirement for producing such input is trained and skillful staff. Hivon et al. [29] argue that there are three major limitations to the use of HTA--organizational, material and scientific. The absence of skilled staff with good knowledge and understanding of HTA is a scientific limitation which makes it difficult to produce context-specific input.

Lack of coordination at the policy-making level of the healthcare system was also found to be problematic. Coordination is generally acknowledged to be an important factor for increasing organizational effectiveness, defined here as a measure of the extent to which a program or sector is successful in achieving its predetermined goals and objectives [30]. Based on this data, the question of who should coordinate who arises and how such coordination is best accomplished. An increase in the number of departments and organizations involved in HT risks leading to escalating complexity and confusion [30], as does involvement of various deputies and departments not only within the MOH but also between the MOH and other organizations such as HIOs. This leads to a wide range of players with different interests and powers, making coordination within and between them difficult.

Another striking finding is the presence of dual practice by physicians in both the public and private sectors, which was described by interviewees as a common pattern. This is very common in many LMICs [31]. Governmental permission for dual practice by physicians is a heavily debated issue. Ferrinho et al. [32], argue that dual practice could result in "predatory behavior" i.e. practitioners prefer their self-gain instead of the legitimate interests of their colleagues or patients. This kind of behavior could lead to generating increasing demand for treatment which in turn would profoundly influence HT utilization. Different potential strategies for limiting dual practice in the public and private sectors utilized in other settings might also be helpful in Iran, e.g. limiting the quantity of services that physicians can perform in the private sector, limiting the income that they can earn from the private sector and raising public salaries [31]. Each strategy selected would need to be implemented consistently to prevent negative consequences of dual practice.

Advertisements by importers and manufacturers of HTs was stated to be an influential factor in relation to adoption and use of HTs in Iran. This is not specific to this setting. Mansfield et al. [33] for example, used Vioxx as an example of how marketing was well beyond science. They also refer to other problems with advertising by exemplifying how marketing of hormone-replacement therapy for preventing cardiovascular disease convinced physicians to use these drugs prior to completion of any clinical trials. In our data, both of these phenomena are present with regard to dissemination and use of MRIs and interferon beta as marketing goes beyond the scientific indications and is said to increase physician and consumer demand. It has also been argued that contexts in which market values dominate leave little room for morality and sound values [34].

The situation described for Iran i.e. having problems with dual practice, the lack of needs assessments, and, in a wider scope, having problems in relation to selection, distribution and use of HTs is also true for some high-income countries [35-38]. Implementing effective policies in these areas remains a challenge.

### Policy implications

A strategic plan, based on needs assessment and priority setting in relation to HT and HTA, is suggested to be determined by the highest authorities of the MOH. An MOH-led overview could lead to the formation of a national coordination body or formulation of a strategic national plan for HT. The presence of a national coordination body could assist the development, implementation, monitoring and evaluation of HT policies and action plans [39]. In many LMICs, even when there are regulations in place, they are not followed strictly [32,40]. It should be emphasized that a national coordination body should not just be a technical advisory section, but should have a mandate to examine compliance with official policy and obstacles to policy implementation. Recent attempts, such as establishing a health reform unit in the MOH, greater orientation towards health system research in universities, development of research in the field of HTA, increasing participation of Iranian researchers in international conferences concerning HT and its assessment and the presence of a 'Health and Treatment Commission' in the Parliament which is able to provide legislative and financial support, all could contribute to make the suggestions above practical.

## Competing interests

The authors declare that they have no competing interests.

## Authors' contributions

MP conceived the study and conducted data collection. MP, CT, SF, HJ, GT participated in the design of the study. MP, CT and AE carried out analysis. All authors participated in writing, reading and approving the final manuscript.
